# Emerging Applications of Versatile Polyaniline-Based Polymers in the Food Industry

**DOI:** 10.3390/polym14235168

**Published:** 2022-11-28

**Authors:** Min-Rui Chia, Sook-Wai Phang, Ishak Ahmad

**Affiliations:** 1 Polymer Research Centre (PORCE), Department of Chemical Sciences, Faculty of Science and Technology, Universiti Kebangsaan Malaysia, Bangi 43600, Selangor, Malaysia; 2 Department of Physical Science, Faculty of Applied Sciences, Tunku Abdul Rahman University of Management and Technology, Setapak, Kuala Lumpur 53300, Malaysia

**Keywords:** polyaniline, intrinsic conducting polymer, active and intelligent food packaging, electronic nose

## Abstract

Intrinsically conducting polymers (ICPs) have been widely studied in various applications, such as sensors, tissue engineering, drug delivery, and semiconductors. Specifically, polyaniline (PANI) stands out in food industry applications due to its advantageous reversible redox properties, electrical conductivity, and simple modification. The rising concerns about food safety and security have encouraged the development of PANI as an antioxidant, antimicrobial agent, food freshness indicator, and electronic nose. At the same time, it plays an important role in food safety control to ensure the quality of food. This study reviews the emerging applications of PANI in the food industry. It has been found that the versatile applications of PANI allow the advancement of modern active and intelligent food packaging and better food quality monitoring systems.

## 1. Introduction

**Figure 1:**
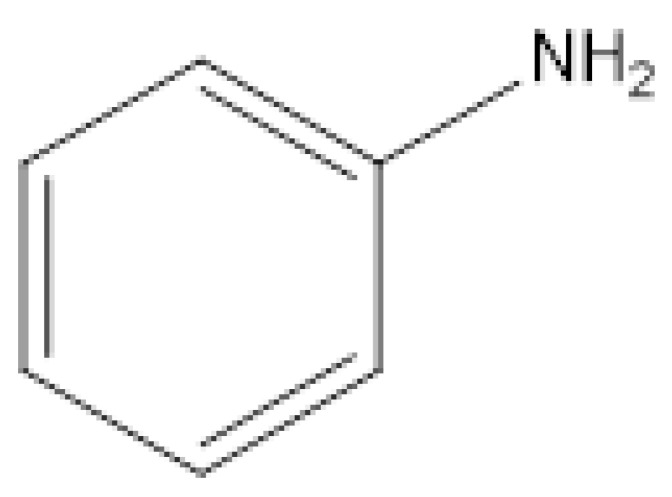
Aniline monomer.

Polyaniline (PANI), as the name suggests, is made up of an aniline monomer (Figure 1). The aniline monomer in PANI is para-substituted, and its chains have a head-to-tail configuration [1,2,3].

**Figure 2:**
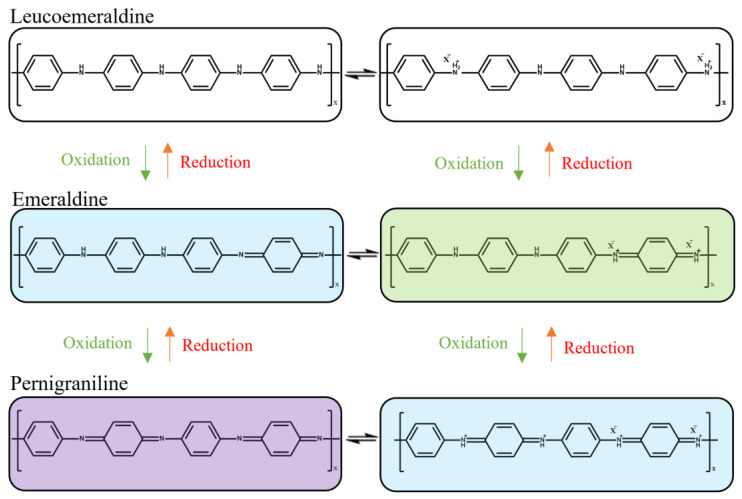
Different oxidation states of PANI.

PANI has orderly arranged chains with alternating phenyl rings and nitrogen-containing groups, as shown in Figure 2 [4,5,6]. It exists in a few stable oxidising states, including pernigraniline, emeraldine salt, and leucoemeraldine, which are violet, green, and yellow in colour, respectively. Emeraldine appears blue when in the form of a base [7,8,9]. The different oxidation states of PANI may be modified through the processes of protonation and deprotonation [10,11,12]. Therefore, the oxidation states of PANI could be affected by changes in pH, leading to a colour change in PANI, thus making it a suitable colourimetric pH sensor [13].

**Figure 3:**
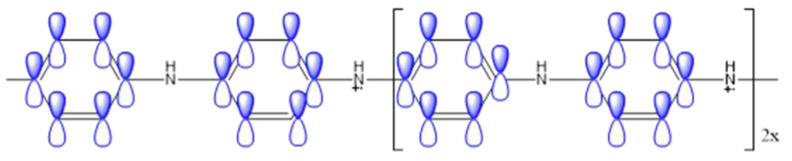
Schematic diagram of the π-system of PANI.

In addition to having various oxidation states with different colours, PANI is an intrinsically electrically conductive polymer (ICP) due to the polaron and bi-polaron charge carriers [14,15,16]. The charge carriers arise from the serrated PANI chain, which lies on one plane on which the π-electron clouds will overlap, resulting in poly-conjugation, as shown in Figure 3 [4,17,18].

**Figure 4:**
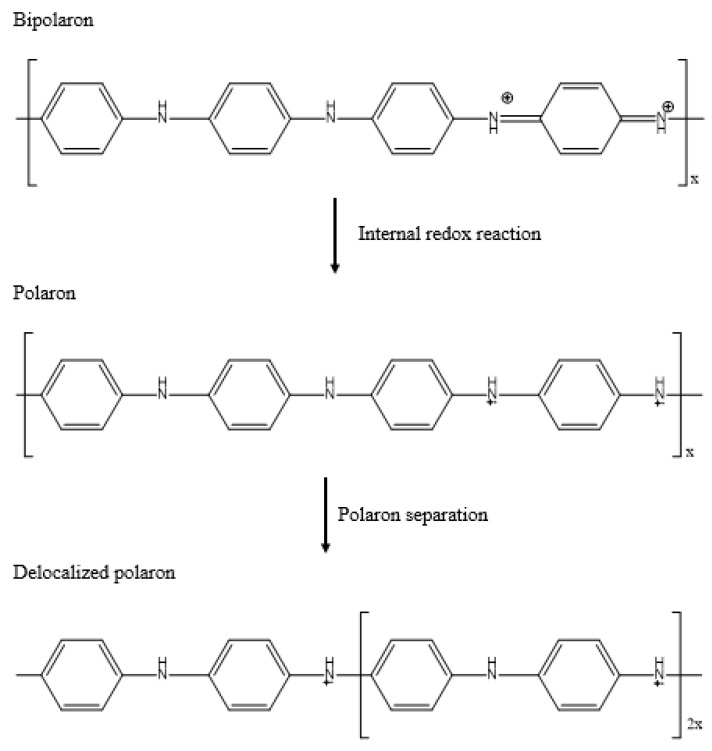
Bipolaron and polaron interconversion in emeraldine PANI.

Poly-emeraldine salt (partially oxidised) is the conductive form of PANI, and it typically has two quinoids out of eight monomers. The nitrogen atoms act as oxidation centres in which the electrons are removed from the nitrogen atoms during oxidation, leading to the generation of positive polarons (Figure 4) [19,20,21]. Therefore, the electrons can move along the central axis of the PANI chain, resulting in the generation of electrical conductivity. In other words, holes will be generated in the highest occupied molecular orbital (HOMO), allowing the migration of charge [1,22,23]. This conducting feature makes PANI a potential material for application in anti-static coating, batteries, light-emitting diodes, and gas sensors [24,25,26,27,28]. In addition, PANI has been proven to impart an abrupt drop in the electrical resistivity of an insulating polymer [29,30,31,32].

**Figure 5:**
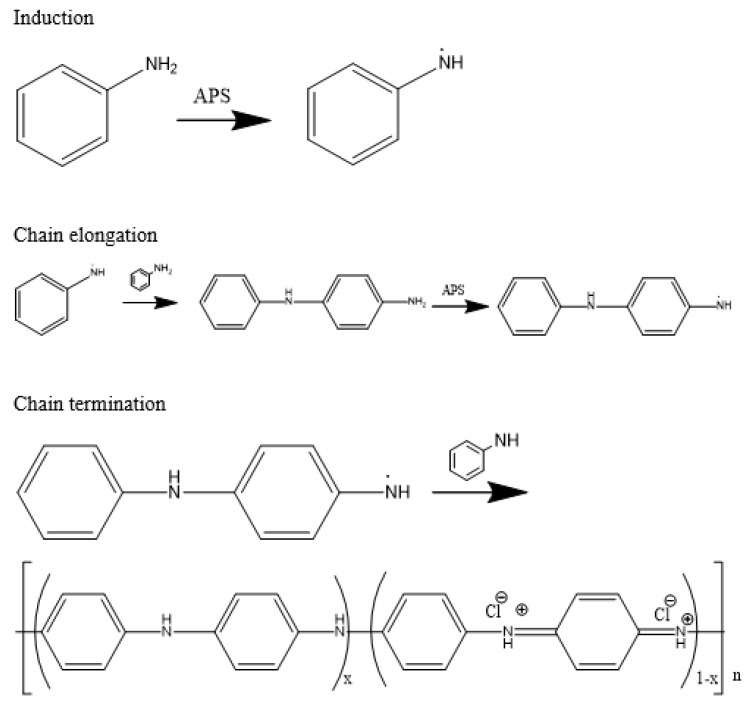
Oxidative polymerisation of aniline into PANI with APS as an initiator.

PANI is usually prepared via oxidative chemical or electrochemical oxidation methods [33,34]. However, for applications in the food industry, the preparation of PANI through oxidative polymerisation is the most popular choice (Figure 5). The polymerisation of aniline can be done through oxidative polymerisation with an oxidant such as ammonium persulfate (APS), hydrogen peroxide, potassium iodate, cerium sulfate, potassium dichromate, potassium ferricyanide, or sodium vanadate, out of which the former is the most common [34,35,36,37,38,39,40,41]. This is because aniline possesses aromatic amine, which tends to be oxidised due to its electron-donating capabilities [42,43]. In addition, polymerisation should be carried out in the presence of strong acids to stabilise the PANI chains [4,44]. Although the terms “doping” and “protonation” are usually applied to describe the interactions between acid and PANI, the acid neither acts as a dopant nor protonates the PANI chains, since it only serves to stabilise the polarons via the interaction of acid anions with neutral nitrogen atoms of PANI [4,45,46]. The pure PANI precipitate is then collected after rinsing it with acid and acetone three to four times to remove unreacted monomers and oxidants. The synthesised PANI powder is in the form of emeraldine salt and has a highly distinguished dark-green appearance [47,48,49].

Furthermore, it is important to understand the toxic aspect of PANI to ensure its safety as a material for its application in the food industry, especially in food packaging. To date, there has been no specific study on the cytotoxicity of PANI-based packaging; nonetheless, there are quite a number cytotoxicity tests that have been carried out on PANI-based polymers for other applications. The toxicity of PANI varies with its content of impurities, oxidation state, size, and shape [50]. In the form of emeraldine salt, PANI exhibits higher cytotoxicity than it does in the form of an emeraldine base. However, when the emeraldine salt’s concentration was kept below 2.5 ppm, it showed no cytotoxicity towards a mouse embryonic fibroblast cell line (NIH/3T3) or embryonic stem cells ES R1 (ESc) [51]. On the other hand, the cytotoxicity of PANI was affected by an acid dopant, where the cytotoxicity increased in the order of PANI–phosphoric acid < PANI–hydrochloric acid < PANI–sulfuric acid < PANI–methanesulfonic acid < PANI–nitric acid, and the most popular HCl-doped PANI did not appear to be cytotoxic at concentrations as high as 20 ppm [52]. PANI nanofibres and nanoparticles were biocompatible at concentrations below 10 and 100 ppm, indicating that PANI nanofibres possessed stronger cytotoxicity [53]. Other PANI-based polymers for applications such as scaffolds, antibacterial materials, biosensors, and drug carriers were also proven to be biocompatible as long as the concentration of PANI was kept beneath the cytotoxicity threshold [54,55,56,57,58].

**Figure 6:**
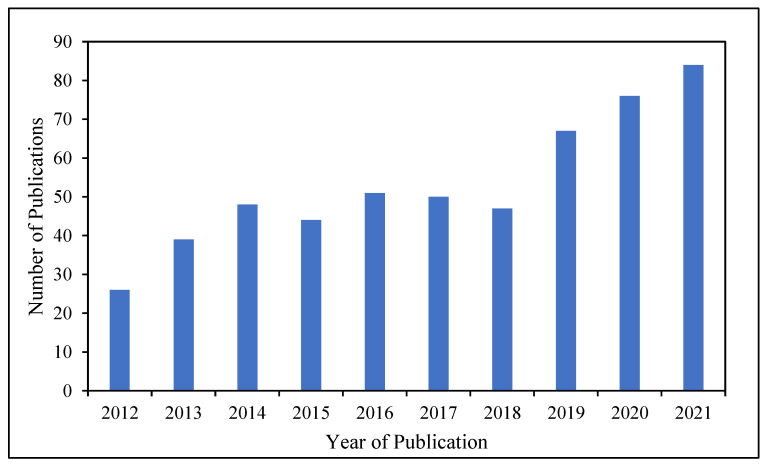
Number of publications regarding applications of PANI in the food industry from 2012 to 2021.

Due to the simple manipulation of its film thickness, the direct deposition of PANI, its large surface area, environmental stability, and its redox conductivity, it has become a very interesting material for various research works, and its applications in the food industry have been well studied. The research on the applications of PANI in the food industry has abruptly increased since the year 2018, indicating that this topic has been gaining researchers’ attention in recent years (Figure 6). However, there is a scarcity of reviews on PANI’s role in the food industry. Therefore, this paper aims to provide a comprehensive review of the applications and importance of PANI in the food industry. Then, there will be a summary of the future perspectives of PANI in the food industry. Thus, with this review, we aim to inspire further in-depth and extensive research on the applications of PANI in the food industry.

## 2. Applications of PANI in the Food Industry

### 2.1. Biodegradable Food Packaging

Biodegradable food packaging is a current trend due to the rising awareness of environmental sustainability, and it is further encouraged by governmental policies, such as the Plastic Tax (European Union), Plastic Packaging Tax (United Kingdom), and Climate Change and Principle-Based Taxonomy (Malaysia). It was recorded that about 584 million tonnes of plastic waste was generated, and packaging waste accounted for over 50% of global plastic waste. Therefore, the replacement of single-use plastics with biodegradable plastics is deemed to overcome plastic pollution in both terrestrial and aquatic ecosystems [59,60]. Biopolymers such as starch, cellulose, and chitosan are widely studied for the replacement of synthetic polymers in food packaging. Nonetheless, these biopolymers are often associated with drawbacks such as hydrophilicity, poor mechanical properties, weak thermal resistance, intrinsic electrical insulation, susceptibility to wet environments, and high cost of production [61,62,63]. PANI is able to improve upon the chemical and mechanical properties of biopolymers so that they can be more feasible for applications in food packaging [64,65,66]. At the same time, studies have shown that biopolymer matrices retain their biodegradability behaviour after the incorporation of PANI [29,67,68,69]. In fact, the addition of PANI improves the biodegradability of low-density polyethylene film (LDPE) via oxo-biodegradation [70]. The combination of PANI with biopolymers not only contributes to the packaging industry, but also has great potential in a broad array of applications, including sensors, supercapacitors, solar cells, dye removers, and optical devices [71,72,73,74,75]. Table 1 shows the improvements of biodegradable polymer matrices in various aspects after the incorporation of PANI.

The hydrophobicity of PANI and the hydrogen linkage interactions between PANI and the matrix reduce the free hydrogen groups for the binding of water molecules [76,77]. Therefore, the incorporation of PANI into chitosan film reduces water solubility and water vapour permeability by approximately 30% and, at the same time, lowers the transmission of light (transparency), allowing the chitosan film to be used in the packaging of foodstuffs that are easily oxidised under light [78]. The strong interaction between PANI and chitosan also improves the processability and tensile strength of biofilms [37,79].

Additionally, the encapsulation of biodegradable polymers by PANI and the cationisation of PANI are used for the enhancement of thermal stability. PANI is able to enhance the thermal stability of whey protein isolate (WPI), starch, cellulose nanofibre (CNF), cellulose nano-whiskers (CNWs), and polylactic acid (PLA) films [6,80,81,82,83]. PANI may also exhibit electrical conductivity and anti-static properties in intrinsically insulating biopolymers, making it a good material for anti-static packaging. Although the electrical conductivity of PANI-based biopolymers is often lowered when compared to that of pristine PANI, nonetheless, they are still suitable for application as anti-static materials. However, the addition of PANI into highly oriented CNF and PLA results in the weakening of tensile strength [29,81]. It is important to note that the poor processability, solubility, and fusibility of PANI are major drawbacks that have to be overcome with suitable blending routes for the preparation of homogenous biodegradable packaging films [84].

**Table 1:** Improvement of biodegradable polymer matrices with the incorporation of PANI.

Matrix	Preparation	Results	Ref
Chitosan	In situ polymerisation	Reduction of water solubility and WVP by 30% Lowering of light transmittance	[78]
		Reduction of electrical resistance Improvement of stiffness and strength by 400%	[79]
WPI	Ex situ polymerisation	Improvement of electrical conductivity up to 0.136 S/m A slight reduction in thermal stability	[80]
Starch	Ex situ polymerisation	Improvement of thermal stability	[6]
Starch/ZnO NPs	Impregnation on ZnO NP	A slight reduction of water solubility and WVP Slight improvement in strength and stiffness	[85]
CNF	In situ polymerisation	Improvement of electrical conductivity up to 4.3 × 10^−2^ S/cm Weakening of strength and stiffness Enhanced thermal stability	[81]
Cellulose acetate	Ex situ polymerisation	Reduction of tensile strength by 27% Reduction of surface resistivity (as low as 7.0 × 10^−9^ Ω/sq)	[86]
CNWs	In situ polymerisation	Improvement of electrical conductivity up to 1.9 S/m Enhanced thermal stability above 500 ℃ by 15% weight loss Lowering of storage modulus	[82]
PLA	Ex situ polymerisation	Weakening of strength, but improved elasticity and stiffness Reduction of surface resistivity (as low as 2.45 × 10^10^ Ω/sq)	[29]
		Improvement of thermal stability at high temperatures (565 ℃) Increased viscosity resulting in greater requirements for the shearing rate Improvement of electrical conductivity (3.42 S/m)	[83]

Abbreviations: WVP, Water vapour permeability, WPI, Whey protein isolate, NPs, Nanoparticles, CNF, Cellulose nanofibre, CNWs, Cellulose nano-whiskers, PLA, Polylactic acid.

### 2.2. Active Food Packaging

**Figure 7:**
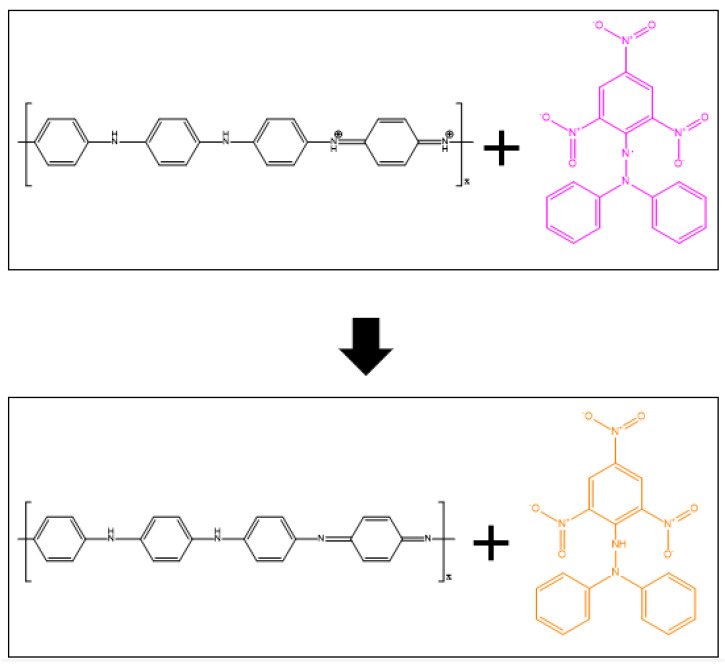
Proposed mechanism of the interaction between PANI and DPPH [50].

PANI is a potential antioxidant material due to its radical scavenging capability [87,88,89]. In the form of emeraldine salt, PANI possesses nitrogen atoms that are capable of electron transfer [90,91,92]. Specifically, the stabilisation of peroxyl radicals depends on the donating ability of the hydrogen atoms [93]. Its antioxidant activity is expected to greatly contribute to active food packaging applications, since it is reported that PANI has a comparable antioxidant activity to that of well-known antioxidants, such as catechin and ascorbic acid [94,95]. The proposed mechanism of the antioxidant activity of PANI against 2,2-diphenyl-1-picrylhydrazyl (DPPH) radicals is shown in Figure 7 [50,96,97].

**Figure 8:**
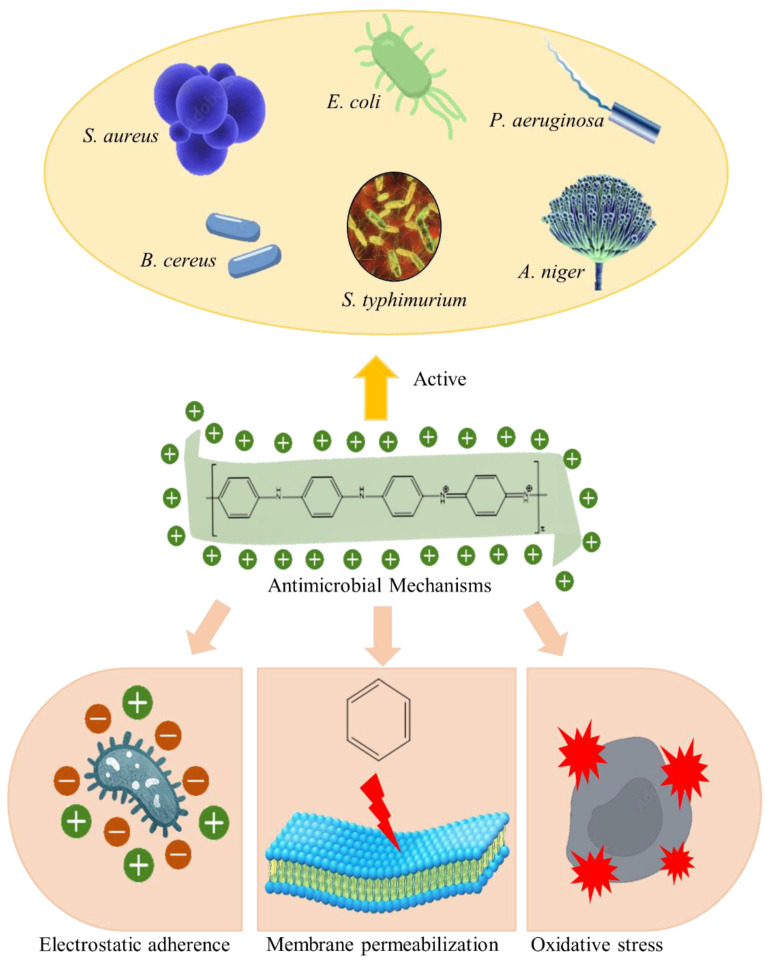
Antimicrobial activity of PANI.

In addition, PANI has been proven to be active against various fungi and bacteria, such as *Aspergillus niger, Escherichia coli*, *Pseudomonas aeruginosa*, *Bacillus cereus*, *Salmonella typhimurium,* and *Staphylococcus aureus* [50,98,99,100]. Its quaternary ammonium structure renders it strong antibacterial and antifungal properties (Figure 8). The biological activity of PANI arises from its electrical conductivity, which could mediate contact on the surface of a bacterial cell via electrostatic adherence [101,102,103]. So, it is more active against Gram-negative bacteria. In addition, the hydrophobic benzene ring on PANI interacts with the membrane core of bacteria, causing membrane permeabilisation. Membrane disruption eventually leads to cell lysis due to the leakage of cellular components and the potential breakdown of the membrane. In addition, PANI may induce oxidative stress on microorganisms through the production of hydroxyl radicals (H_2_O_2_), leading to the Fenton reaction. In this reaction, free ions accelerate the formation of H_2_O_2_, causing cell destruction [50].

Several studies on the antioxidant and antibacterial activities of PANI have been conducted for the purpose of active food packaging (Table 2) [37,104,105]. PANI was proven to improve the mechanical properties, electrical conductivity, and antimicrobial activity of pure chitosan film [79]. PANI-coated PMMA/CNC showed 45% inhibition of DPPH after 240 min and was active against *B. cereus* and *S. typhimurium* [106]. On the other hand, chitosan/PANI exhibited slight antioxidant strength. PLA/PANI film strengthened with CuO and ZnO also performed well as an antibacterial film against *S. aureus* and *E. coli*. The film was proven to slow down the microbial growth (total aerobic bacteria and acidophilus bacteria) in orange juice, thus preserving the quality of orange juice and resulting in a longer life span [107].

### 2.3. Intelligent Food Packaging

Microbial growth and metabolism are major causes of food spoilage, and they result in the formation of amines, sulfides, alcohols, aldehydes, ketones, and organic acids with unpleasant and unacceptable off-flavours [115,116,117]. Trimethylamine (TMA), dimethylacetamide (DMA), decomposed urea, and amino acid are released in the form of ammonia during food spoilage by bacteria [118]. Therefore, ammonia and TMA are common marker gases for indicating food spoilage [119,120,121]. They are usually released during the spoilage of high-protein foods, but can also be released by spoiled vegetables, including spinach, seaweed, and corn [122,123].

Moreover, *E. coli* is a very common food-borne bacterial pathogen found in the gut of cattle. *E. coli* often leads to food-borne illnesses such as haemolytic uremic syndrome and haemorrhagic colitis. In addition, due to the extreme climatic change, COVID-19 pandemic, and Russia–Ukraine war, people are alarmed about global food insecurity and are concerned about solutions for tackling food waste [124]. According to the Food Waste Index Report 2021 by the United Nations (UN), in 2019, approximately 931 million tonnes of food waste (17% of global food production) was generated. Therefore, it is crucial to develop a rapid and reliable technique for the detection of *E. coli* in foodstuffs. An effective and simple colourimetric sensor can be utilized to detect the presence of these gases within food packaging to monitor the quality of perishable foods in a real-time manner and reduce food waste [119].

**Figure 9:**
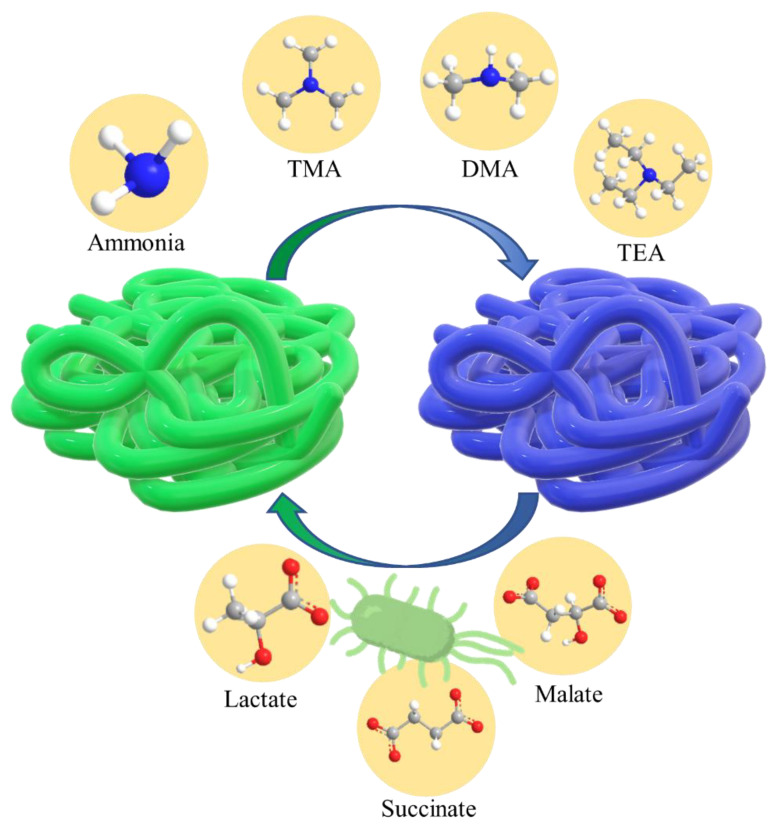
PANI as a colourimetric sensor for the indication of food freshness.

As a stimulus-responsive polymer, PANI is able to conduct electricity and is sensitive to pH changes through protonation and deprotonation of its central axis. It has been widely studied as a colourimetric indicator of food freshness (Figure 9) [125,126,127]. Starch/PANI film was studied as an ammonia sensor for the purpose of the indication of food spoilage. During food decomposition, ammonia vapour was released, and its interaction with PANI changed it from a green emeraldine salt into a blue emeraldine base. This starch/PANI colourimetric sensor exhibited a limit of detection (LOD) as low as 245 ppm and a relative standard deviation (RSD) of 8.72% [6]. PANI was also specifically applied to food samples as a real-time food freshness indicator. PANI/TPE showed a good linear response to TVB-N within the concentration range of 25.2 mg to 100 g and was able to indicate the spoilage of live red drum (*Sciaenops ocellatus*) fish through its colour change from emerald green to peacock blue [128]. A similar study was also performed to evaluate the freshness of tilapia, where doped PANI film acted as a colourimetric sensor, even under chilled conditions (4 ℃), and was able to be recycled up to three times [129]. On the other hand, *E. coli* in milk and butter was detected by PANI through colour changes due to its interaction with the acidic product of *E.coli*’s glycosidic pathways, such as succinate, acetate, lactate, and malate [130].

Furthermore, the changes in PANI’s electrical conductivity due to gases released during food spoilage make it a simple yet effective food freshness indicator (Table 3). This is due to the flexible polar bond rotation, which eventually modifies the PANI chains through the structure of complex charge transmission, resulting in AC conductivity [131]. There was a more recent study in which a PANI/silver nanowire/silk composite was applied as a resistometric microsensor for the detection of TMA, and it was capable of indicating the freshness of pork. The high sensitivity (LOD: 3.3 µg/L), good stability, and repeatability (up to five cycles) made this composite a great potential freshness indicator for pork [132]. In addition, in a PLA/ZnO/CuO film, PANI played a significant role in the estimation of the shelf life and expiration date of orange juice, where it yielded an accuracy higher than 90%. As the spoilage of the juice began, gases were released, and they applied pressure to the smart film as they accumulated in the packaging. The pressure applied caused a change in the electrical conductivity of the film, thus indicating food spoilage [107]. However, the greatest challenge of PANI in intelligent food packaging is that the electroconductivity of PANI is easily affected by humidity, leading to the false detection of target molecules [133]. Moreover, the lack of an evaluation of its toxicity and the imposed risk of the migration of substances are stumbling blocks on the path toward PANI-based food packaging [134,135].

### 2.4. Food Safety Control

**Figure 10:**
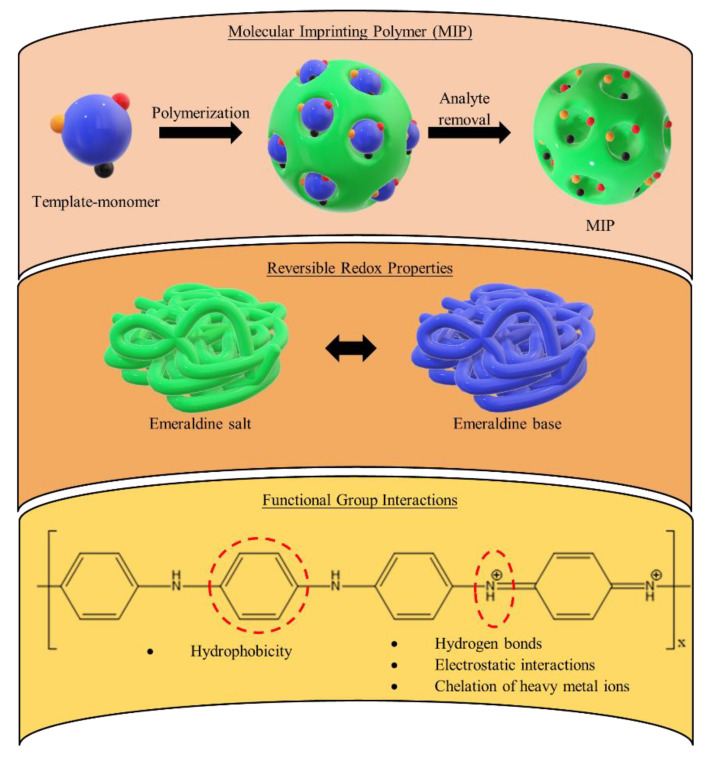
Mechanisms of PANI-based devices for the detection and/or extraction of contaminants in food samples.

Antibiotic contamination from the wastes of hospitals, the pharmaceutical industry, and human and animal excretion often occurs in an aquatic environment, where prolonged unintended exposure towards these antibiotics promotes antibiotic resistance in humans [144,145,146]. Therefore, a PANI-nanofibre-coated U-bend optical-fibre-based sensor is a useful tool for monitoring β-lactam antibiotics in food. During the enzymatic hydrolysis of β-lactam, protons and acidic by-products, such as penicillinoic acid (from penicillin) and ampicilloic acid (from ampicillin), are released, leading to a pH change that converts PANI from the form of an emeraldine base into that of an emeraldine salt, which is measured by the increase in wave absorbance at 435 nm. This optical fibre sensor is able to detect penicillins and cephalosporins in food, including milk, chicken, and water, with an LOD as low as 0.18 nM [147]. PANI nanowires have also been synthesised into molecular-imprinted (MIP) sensors, where the nanowires were electro-polymerised onto a gold electrode and then imprinted with chloramphenicol as a molecular template (Figure 10). It turned out that a PANI MIP sensor was able to detect chloramphenicol at levels as low as 10^−7^ mM [148]. In addition, magnetic mesoporous PANI coated with hydrophilic monomers and casein for solid-phase extraction coupled with HPLC was able to simultaneously determine a wide array of antibiotics in milk samples with recoveries as close as 100%. These included doxycycline, oxytetracycline, trimethoprim, and penicillin G [149]. Similarly, PANI/GO was used for the electrochemically controlled SPME of antibiotics—specifically, tetracyclines—in milk samples, with recoveries ranging from 71% to 104% [150].

Non-steroidal anti-inflammatory drugs (NSAIDs) are common veterinary medicines, and NSAID residues in animal-originating foodstuffs adversely affect human health, causing cardiovascular diseases, gastrointestinal ulceration, kidney toxicity, and platelet aggregation inhibition [151]. Solid-phase extraction (SPE) with a PANI/PAC nanofibre mat was used in the extraction of NSAIDs, as the amino and benzene ring groups of PANI rendered it a strong affinity towards NSAIDs via a hydrogen bond, π–π interaction, its acid–base function, and its hydrophobicity (Figure 10). This showed practical feasibility with meat and egg samples and was able to detect a wide array of NSAIDs (ibuprofen, naproxen, diclofenac, carprofen, ketoprofen, tolfenamic acid, and salicylic acid) with LODs and recoveries in the ranges of 0.6–12.2 µg kg^−1^ and 85.18–107.31%, respectively [152]. Other similar studies on the extraction of NSAIDs using PANI have also been done in recent years (Table 4) [153,154,155].

Heavy metal ions, such as lead, chromium, cobalt, and copper, are severely toxic to human health, even at low dosages [156,157,158]. In order to separate, enrich, and detect metal ions at trace levels, SPE is one of the most selective and sensitive techniques. Electrically or magnetically assisted SPE eliminates the use of eluent by changing the surface of the conducting sorbent, thus simultaneously improving the extraction efficiency. Due to its reversible redox and electroactivity properties, PANI is an excellent candidate as the conducting sorbent of electrically assisted SPE [159]. Moreover, the abundance of imine and amine functional groups in PANI also enhances the adsorption of heavy metal ions (Figure 10) [160,161]. A PANI nanofibre–graphene oxide (GO) sorbent was developed to extract Pb^2+^, and it achieved an LOD, RSD, and reproducibility of 0.04 µgL^−1^, 1.97%, and 2.51%, respectively [162]. Another similar study was performed by using SiO_2_-coated GO/PANI/Polypyrole (PPy) in magnetic SPE of Cr(III) and Pb(II) with LODs of 4.808 and 3.401 ngL^−1^, respectively [156]. In addition to SPE, free-standing PANI composites have also been studied in the adsorption of heavy metals from aqueous samples [163,164,165,166]. Although volume changes in PANI—either swelling or shrinking—may occur during electrochemical cycling, thus adversely affecting its stability, most of the PANI-based sensors reported for food safety control have exhibited reliable stability with good reproducibility [154,157,162].

**Table 4:** Detection and/or extraction of contaminants such as antibiotics, NSAIDs, and heavy metal ions by PANI-based devices.

Contaminant	Device	Target	Food	Ref
Antibiotic	β-Lactamase immobilised on PANI-coated optical fibre	β-lactam antibiotics	Packaged milk, cow milk, buffalo milk	[147]
	SPME based on Cu/PANI/GO coupled with HPLC-UV	DOX, OXY, TET	Water, pasteurised bovine milk	[150]
	MSPE based on RA/MMPANI/HM/CAS coupled with HPLC	DOX, OXY, TPM, PEN-G	Milk	[149]
	PANI NW-based MIP deposited on a gold electrode	CHL	-	[148]
	PANI/GO/QD-based MIP probe	LOM	Milk, chicken meat, egg	[167]
NSAID	SPE based on PANI NFM coupled with UPLC-MS/MS	IBU, NAP, DC, CPF, KTP, TLF, SA	Meat, egg	[152]
	CS/PANI/ZnAl-LDH	NAP	-	[154]
	Au/PANI-based MIP membrane	IBU	-	[153]
	SPME based on graphene/PANI coupled with IMS	MFA, IBU	-	[168]
Heavy metal ions	MSPE based on PANI-coated magnetic NPs coupled with FAAS	Co(II)	Soft drinks, spices, vegetables, water	[169]
	PAN/PANI membrane	Pb(II), Cr(VI)	Water	[163]
	Electrochemically assisted SPE based on PANI NF/GO coupled with FAAS	Pb(II)	Juices (peach, orange, grape), water (tap, mineral)	[162]
	MSPE based on SiO_2_-magnetic GO/PANI/PPy coupled with ICP-MS	Cr(III), Pb(II)	Water, rice, milk, wine	[156]
	SPE based on nanostructured PANI coupled with FAAS	Cu(II), Pb(II)	Shrimp, crab, fish, apple, tomato, mushroom, potato, water	[170]

Abbreviations: SPME, Solid-phase microextraction, GO, Graphene oxide, HPLC, High-performance liquid chromatography, DOX, Doxycycline, OXY, Oxytetracycline, TET, Tetracycline, MSPE, Magnetic solid-phase separation, RA, Restricted access, MM, Magnetic mesoporous, HM, Hydrophilic monomers, CAS, Casein, TPM, Trimethoprim, PEN-G, Penicillin G, NW, Nanowire, MIP, Molecular-imprinted polymer, CHL, Chloramphenicol, QD, Quantum dots, LOM, lomefloxacin, NFM, Nanofibre mat, UPLC-MS/MS, Ultraperformance liquid chromatography–tandem mass spectrometry, IBU, Ibuprofen, NAP, Naproxen, DC, Diclofenac, CPF, Carprofen, KTP, Ketoprofen, TLF, Tolfenamic acid, SA, Salicylic acid, CS, Carbon sphere, LDH, Layered double hydroxides, IMS, Ion mobility mass spectrometry, MFA, mefenamic acid, NPs, Nanoparticles, FAAS, Flame atomic absorption spectrometry, PAN, Polyacrylonitrile, NF, Nanofibre, PPy, Polypyrrole, ICP-MS, Inductively coupled plasma mass spectrometry, SPE, solid-phase extraction.

### 2.5. Electronic Noses

The discrimination of aromas is important for the determination of the freshness, quality, and safety of food. The π-conjugated PANI chains capable of electron delocalisation make it suitable for application as a sensitive layer for gas sensors. A HCl-doped PANI electronic nose system’s potential effectiveness was proven in detecting and distinguishing several aromas, and it showed the best sensitivity towards grapes (112%) [171]. In another study, an electronic nose with PANI-layered gold-interdigitated microelectrodes (IDEs) was also able to analyse artificial aromas found in gummy candies by detecting aromas with concentrations as low as 900 ppb, as it had good reversibility (97.6%) [172,173]. Another similar study in situ involved the polymerisation of PANI onto a graphite-interdigitated electrode to monitor the release of aromas (apple, strawberry, and grape) from gummy candies. It showed that an electronic nose with camphor sulfonic acid (CSA)-doped PANI had the best sensitivity towards artificial aromas [174]. On the other hand, a PANI/functionalised single-wall carbon nanotube was developed into an electronic nose for the detection of ammonia vapours to monitor the freshness of beef [175].

## 3. Conclusions and Future Perspectives

In a nutshell, this review included some remarkable applications of PANI in the food industry from the last few years. The ability of PANI to improve the chemical, physical, and mechanical properties of biopolymers makes it a good material for food packaging. To make it even more valuable, the antioxidant and antimicrobial properties held by PANI allow it to perform specifically as an active and intelligent food packaging material. In addition, PANI is able to respond sensitively and selectively towards various analytes, including ammonia, TMA, antibiotics, NSAIDs, heavy metals, microbial growth, and artificial aromas; thus, it can be readily applied in sensors and electronic noses. The performance of PANI can also be enhanced through the incorporation of metal oxides and nanoparticles such as nanocellulose.

Nonetheless, in order to enhance the feasibility of using PANI in the food industry, thorough toxicology tests should be included to identify the hazards of using PANI among humans and animals. These should include the comprehensive assessment of toxicology profiles, mutagenic potential, genetic toxicology assays, and structure–activity relationship analysis (SAR). Since the polymerisation of PANI involves the usage of harmful chemicals and the generation of unwanted side-products, analyses should be performed to assess the presence of impurities in PANI and to ensure that these contaminants are kept below the safety thresholds. Moreover, overall and specific migration tests should be performed to identify and quantify the transition of PANI from its matrix into food substances. This is to ensure that food safety is not compromised with the addition of PANI as a food contact substance. Moreover, it is well known that PANI exhibits redox properties. Ideally, PANI-based active and intelligent food packaging should be renewable; however, there is lack of similar tests on active and intelligent packaging derived from PANI. Therefore, the renewable antioxidant, antimicrobial, and sensing features of PANI in food packaging should be explored later on. In conclusion, PANI has great potential in the food industry, and further research can be done so that it is feasible to commercialise PANI for its applications in the future.

## Figures and Tables

**Table 2:** Some examples of antioxidant and antimicrobial applications of PANI and PANI composites.

Composites	Antioxidant Properties	Antimicrobial Towards	Food	Ref
Chitosan/PANI	√	-	-	[78]
CS/PANI-ZnO NP	-	*S. aureus* *E. coli*	-	[85]
PANI-(PMMA/CNC)	√	*B. cereus* *S. typhimurium*	-	[106]
Chitosan/PANI	-	*A. niger* *E. coli*	-	[79]
PANI	-	*S. aureus* *E.coli*	-	[108]
Cellulose/PANI				
Chitosan-ZnO/PANI	-	*S. aureus* *P. aeruginosa*	-	[109]
Dextrin/PANI	√	-	-	[110]
PCL/NF PANI	√	*S. aureus* *E. coli*	-	[111]
LLDPE/NR PANI	√	*S. aureus*	Fish oil	[112]
EC/PANI	√	-	Fish oil	[113]
PET/NR PANI	√	-	-	[114]
PLA/PANI/ZnO/CuO	√	*S. aureus* *E. coli*	Orange juice	[107]

Abbreviations: CS, Corn starch, NP, Nanoparticles, PMMA, polymethyl methacrylate, PCL, Poly-ε-caprolactone, NF, Nanofibrous, LLDPE, Linear low-density polyethylene, NR, Nanorod, EC, Ethylcellulose, PET, Polyethylene terephthalate, PLA, Polylactic acid.

**Table 3:** Applications of PANI as a food freshness indicator.

Method	Label	Target	Food	Ref
Colourimetric	PANI-Pec	E. coli	Milk and butter	[130]
	Starch/PANI	NH_3_	-	[6]
	PANI/TPE	Spoilage gas	Red drum fish	[128]
	PANI	TMA	Blue marlin fish	[126]
	PANI	NH_3_ and DMA	Tilapia	[129]
	PANI-Pec NP	E. coli	Tap water	[136]
	PANI-PSS	TEA	-	[137]
	PANI	NH_3_	Milkfish	[138]
Resistometric	PLA/PANI/CuO/ZnO	Spoilage gas	Orange juice	[107]
	PANI/Ag NW/Silk	TMA	Pork	[132]
	PTS-PANI	NH_3_, putrescine, and cadaverine	Beef, pork, fish, and chicken meat	[139]
	TiO_2_-PANI/SFF	NH_3_	Pork	[140]
	PANI-PI	NH_3_	Meat	[141]
Potentiometric	PANI NF/PET	pH	Milk & apple	[142]
Conductometric	HEC/PANI	pH	Milk	[143]

Abbreviations: Pec, Pectin, TPE, Tetraphenyl ethylene, TMA, Trimethylamine DMA, Dimethylamine, NPs, Nanoparticles, PSS, Poly(sodium 4-styrenesulfonate), TEA, Triethylamine, PLA, Polylactic acid, NW, Nanowire, PTS, p-Toluene sulfonate hexahydrate, SFF, Silk fibroin microfibre, PI, Polyimide, NF, Nanofibre, PET, Polyethylene terephthalate, HEC, Hydroxyethyl cellulose.

## Data Availability

The data presented in this study are available on request from the corresponding author. The data are not publicly available due to copyright issue.
